# Large Area Microfluidic Bioreactor for Production of Recombinant Protein

**DOI:** 10.3390/bios12070526

**Published:** 2022-07-14

**Authors:** Natalia Bourguignon, Paola Karp, Carolina Attallah, Daniel A. Chamorro, Marcos Oggero, Ross Booth, Sol Ferrero, Shekhar Bhansali, Maximiliano S. Pérez, Betiana Lerner, Gustavo Helguera

**Affiliations:** 1 Centro IREN, Universidad Tecnológica Nacional, Haedo B1706EAH, Provincia de Buenos Aires, Argentina; nbourgui@fiu.edu (N.B.); daniel.chamorro89@gmail.com (D.A.C.); maxperez@fiu.edu (M.S.P.); 2 Department of Electrical and Computer Engineering, Florida International University, Miami, FL 33174, USA; sbhansa@fiu.edu; 3 Laboratorio de Biotecnología Farmacéutica, Instituto de Biología y Medicina Experimental (IBYME-CONICET), Ciudad de Buenos Aires C1428ADN, Argentina; paoka_27@hotmail.com (P.K.); sool.ferrero@gmail.com (S.F.); 4 Centro Biotecnológico del Litoral, Laboratorio de Cultivos Celulares, Facultad de Bioquímica y Ciencias Biológicas, Universidad Nacional del Litoral (UNL), CONICET, Santa Fe S3000ZAA, Provincia de Santa Fe, Argentina; attallah@fbcb.unl.edu.ar (C.A.); moggero@fbcb.unl.edu.ar (M.O.); 5 Roche Sequencing Solutions, Inc., Pleasanton, CA 94588, USA; ross.booth@roche.com

**Keywords:** microfluidic bioreactor, large-area, cell culture, monoclonal antibody, anti-hIFN-α2b, antibody activity

## Abstract

To produce innovative biopharmaceuticals, highly flexible, adaptable, robust, and affordable bioprocess platforms for bioreactors are essential. In this article, we describe the development of a large-area microfluidic bioreactor (LM bioreactor) for mammalian cell culture that works at laminar flow and perfusion conditions. The 184 cm^2^ 32 cisterns LM bioreactor is the largest polydimethylsiloxane (PDMS) microfluidic device fabricated by photopolymer flexographic master mold methodology, reaching a final volume of 2.8 mL. The LM bioreactor was connected to a syringe pump system for culture media perfusion, and the cells’ culture was monitored by photomicrograph imaging. CHO-ahIFN-α2b adherent cell line expressing the anti-hIFN-a2b recombinant scFv-Fc monoclonal antibody (mAb) for the treatment of systemic lupus erythematosus were cultured on the LM bioreactor. Cell culture and mAb production in the LM bioreactor could be sustained for 18 days. Moreover, the anti-hIFN-a2b produced in the LM bioreactor showed higher affinity and neutralizing antiproliferative activity compared to those mAbs produced in the control condition. We demonstrate for the first-time, a large area microfluidic bioreactor for mammalian cell culture that enables a controlled microenvironment suitable for the development of high-quality biologics with potential for therapeutic use.

## 1. Introduction

Microfluidics provides promising platforms for lab-on-a-chip (LOC) applications and enables the miniaturization of basic conventional biological or chemical laboratory operations. Laboratory technology in a microfluidic device has been well accepted by the biological and medical research communities as a promising tool for engineering microenvironments at the molecular, cellular, and tissue levels [1]. Unlike conventional static approaches, microfluidic-based cell cultures can maintain well-defined cell culture conditions and, more importantly, allow cells to be continuously supplied with fresh media containing oxygen and nutrients while removing contaminants [1,2,3].

For adherent cells, the hydrodynamic control of the microenvironment affects not only the rate of nutrient delivery and replenishment but also defines the rate of dispersion of extracellular molecules and the mechanical stress exerted by the shear force on the cells adhering to their substrate [4]. Furthermore, the small volumes of the microchannels increase the concentration of secreted proteins [5]. When cells are introduced into a microfluidic channel, they exhibit a rounded shape. They settle and adhere to the channel’s surface by electrostatic attraction or by proteins on the surface of the microchannel through integrins [6]. Once cells are attached, they flatten and secrete extracellular matrix proteins and begin to proliferate. Cell adhesion is an important step to initiating the release of signals that regulate cell differentiation, the cell cycle, migration, and survival [7].

The fluid flow in the channels produces shear stress on the cells, which competes with the forces of adhesion between the cells and their substrate [8]. Hemodynamic flows present high shear stress (>1 Pa), which could induce a stress response in the adherent cells. This can lead to the alteration of the adherence properties of the cells that may lead to a rounded shape, surface separation, and changes in adhesion protein expression. The reconfiguration of the cytoskeleton could cause cell apoptosis in certain scenarios [4,9]. Whereas at low shear stress (<1 Pa), as in the interstitial, extravascular flow in the body, and hydrodynamic forces constitute a mechanotactic signal that can lead to the movement of a cell and affect its differentiation [4]. The impact of shear stress on cells varies by cell type [10], but up to 0.65 Pa, it has no biological impact on most cells. In culture devices at a flow rate of 500 µL/min, the shear stress is estimated to be 10^−4^ Pa, several orders of magnitude below that observed to affect shear stress on cells. Therefore, at that flow rate, shear stress is not expected to have any effect on cell viability.

For cell culture, microfluidic techniques are powerful tools because of their ability to generate homogeneous and controllable complex microenvironments for the cells in microchannels [11]. In addition, microfluidic device cell culture methods have several advantages, such as cost-effectiveness, reproducibility, low volume requirements, simple and accurate visualization, speed of testing, versatility of designs, and portability [12]. In particular, for the expression of recombinant proteins intended for therapeutic use, these miniaturized microfluidic technologies will provide increased volumetric productivity, good quality product, shorter response time, reduced sample volumes, power requirements, and manufacturing costs for process optimization [13,14].

The development of monoclonal antibody (mAb) technology has enabled the generation of therapeutic antibodies with the ability to selectively target antigens and are used for the treatment of serious diseases, including autoimmune disorders and cancer [15,16,17]. With the increasing number of mAbs entering various stages of development, there is an interest in innovative solutions to deliver this pipeline. The fact that mammalian cells are relatively difficult to work with due to factors such as medium complexity, serum requirement, low yield, and shear sensitivity has important implications in terms of manufacturability and scalability of mAbs [18]. The commercial production of mAbs and other biotherapeutic products is currently based on synthesis in 5 to 20 m^3^ bioreactor tanks with mammalian cells by shaking suspension culture operated in a fed-batch or perfusion mode [19,20,21]. The commercial production of mAbs in stirred tanks faces challenges related to product and process quality, such as demands for higher productivity, glycosylation control, reproducibility, as well as other process controls [19,21,22]. Most of these challenges are related to the great temporal and spatial variability of the intrinsic conditions of the fermenters. One way to improve the understanding and control of mAb production in bioreactors is to downscale the system through miniaturization in the form of microfluidic devices [23].

There are few studies on the use of LOC-type devices to produce recombinant proteins [24]. In previous work, we have demonstrated the feasibility of the production of an anti-hIFN-α2b recombinant mAb with Chinese hamster ovary, CHO, and human embryonic kidney HEK-293 cells in standard-size microfluidic devices with a final volume of 32.22 µL [25]. It was possible to increase the concentration of the antibodies up to 7.3 times higher (1120 pg of mAb per cell) than that obtained by standard culture methodologies (using T-flasks). Moreover, the functional activity of the antibodies produced in the microdevice (neutralization of the cellular cytokine signaling) did not present a significant difference from those obtained in the control culture. In this context, the main objective of the present work is to scale up the production of recombinant proteins in the large-area microfluidic bioreactor (LM bioreactor). To achieve that, we developed the LM bioreactor with a novel fabrication technique [26] and we validated the technology by producing and characterizing mAbs.

The methodology used here allows the manufacture of microfluidic devices with a larger area and volume than the standard devices in this field and was developed by our group using a photopolymer flexographic master mold (Fmold) [26]. This photopolymer enables the fabrication of master microfluidic templates with dimensions up to 1270 × 2062 mm^2^, frame heights between 53 and 1500 μm, precise dimensions, and a minimum dot reproduction of 10 μm. A minimum structure size of 25 μm is possible to achieve, allowing the integration of multiple laboratory functions and detection systems into a single layer. The reproduction fidelity, stability, and durability of the Fmold technique combined with the reusable epoxy resin mold method (ERmold) were tested by producing up to 50 polydimethylsiloxane (PDMS) replicas, and the surface morphological characteristics of the Fmold and PDMS replicas were preserved [26]. The Fmold offers a good alternative to conventional fabricating photo resin microfluidic devices fabricated on silicon wafers. 

Here, we show the advantages and limitations of scaling up the production of recombinant proteins in microfluidic-based bioreactors by culturing the producing cell line and characterizing the functionally of the anti-hIFN-α2b mAb produced in this novel device. 

## 2. Materials and Methods

### 2.1. LM Bioreactor Design and Fabrication

Layout Editor software was used for the design of the bioreactor microchannels architecture [27]. The LM bioreactor design involves 32 identical microchannels (1800 µm width), one inlet, and one outlet. The channel height is 200 µm, the channel surface area is 184 cm^2^, and the total internal volume is 2.8 mL in a 20 cm × 30 cm^2^ area. The design has curved wave channels in the connections to avoid the formation of bubbles and the accumulation of cells in straight corners [28].

**Figure 1:**
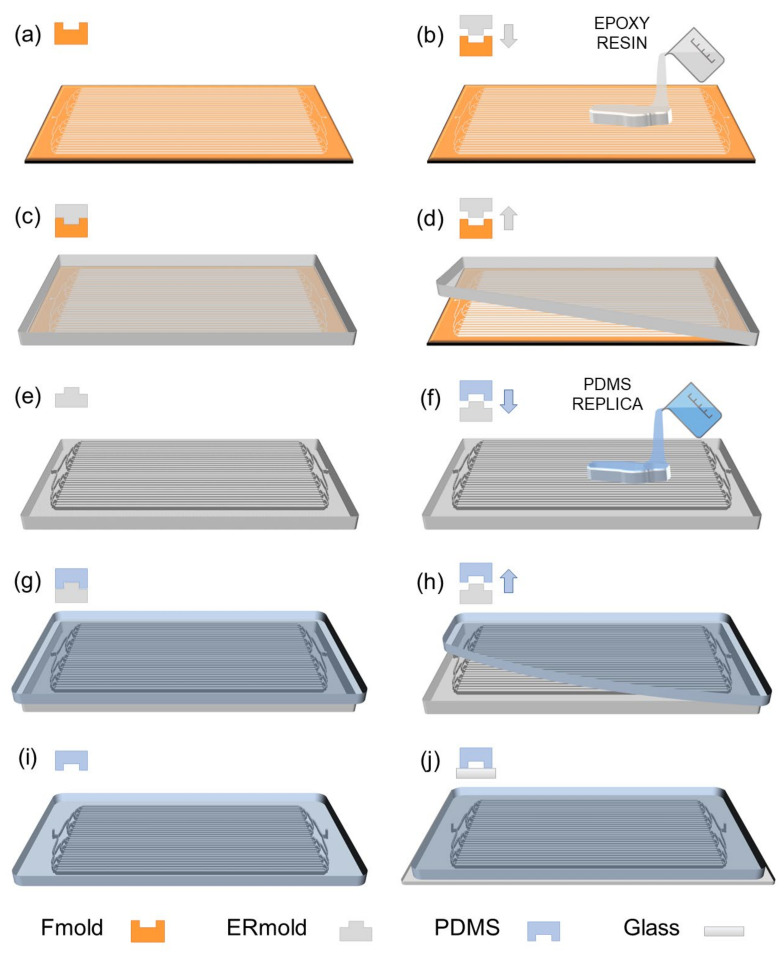
PDMS LM bioreactor device fabrication: (**a**) Female patterned photopolymeric flexographic master mold (Fmold); (**b**) the epoxy resin layer is cast on the female Fmold; (**c**) the epoxy resin is cured at 25 °C for 72 h; (**d**) the cured epoxy layer is peeled off; (**e**) the male ERmold is complete; (**f**) the PDMS replica is cast on the male ERmold; (**g**) the PDMS layer is cured at 40 °C overnight; (**h**) the female PDMS replica is peeled off; (**i**) The fluidic connection entry and exit ports are punched on the female PDMS; (**j**) the PDMS replica with the design of the bioreactor microfluidic channels is irreversibly bonded by plasma exposure to a glass wafer.

The LM bioreactor was built using polydimethylsiloxane (PDMS; Sylgard 184, Dow; Corning) on the top and glass on the base. Three main steps comprise the PDMS microfluidic device manufacturing process: (1) Photopolymer flexographic master mold (Fmold) fabrication, (2) manufacture of a reusable epoxy resin male mold (ERmold) from the FMold by a copy of the mold, and (3) PDMS replica from ERmold features (Figure 1). The complete methodology was developed by the group and has been described in previous works [29,30,31].

*Photopolymer flexographic master mold (Fmold):* Eastman Kodak supplied the photopolymer Flexcel NX and Thermal Imaging Layer (TIL) to fabricate the Fmold [31,32]. The fabrication steps of the Fmold and ERmold have been described in previous works [29,33]. Briefly, the microchannels architecture was designed with Layout editor software; this design was transferred to the TIL with an infrared laser source of 2400 ppi [32] and covered onto the photopolymer plate. Later, the photopolymer plate was exposed to UVA on the reverse and front sides, and the TIL was removed [26]. Next, the photopolymer plate was washed with solvent PROSOL N-1 at 360 mm/min and dried in an oven for 30 min at 50 °C. Finally, the photopolymer plate was exposed to UVC and UVA on the front side. This mold was coded as Fmold. Before use, the Fmold was placed in an oven at 100 °C for 12 h and then was treated in a vacuum chamber for 1 h at 25 °C, followed by cleaning in 70% (*v/v*) ethanol solution in an ultrasonic bath for 7 min, dried at 40 °C for 10 min and cleaned in a nitrogen stream.

*Epoxy resin mold (ERmold):* A commercially available epoxy resin and curing agent (Cristaltack, Novachem S.A., Villa Martelli, Argentina) were mixed in a 2:1 weight ratio by hand-stirring for 3 min and ultrasonically treated using a bath-type sonicator (TESTLAB Ultrasonic Cleaner, Bernal, Argentina) for 7 min to remove air bubbles. Then, the mixture was poured onto the Fmold and cured at room temperature for 72 h. After curing, the epoxy resin mold was peeled off from the Fmold to form the male mold; this mold is referred to as ERmold. 

*PDMS microdevice:* PDMS was mixed with a curing agent at a ratio of 10:1 (200 g total). The mixture was placed under a vacuum to remove air bubbles for 1 h. After this, the mixture was poured into the ERmold and cured in an oven at 40 °C overnight. Finally, the device was peeled off the mold, cut, and 1 mm diameter fluidic connection ports were punched using a biopsy puncher (Integra Miltex^®^Ted Pella, Inc., Redding, CA, USA). The device was then irreversibly bonded to glass after exposure to a high-frequency generator (BD 10AS, Chicago, IL, USA) for 30 min. The glass was previously treated with Gorilla Nano Glass Liquid Universal Nanotechnology Screen Protector (Nano Hi tech, Guangzhou, China) to increase its hardness. Stainless steel catheters and tubing (0.8 mm diameter) with syringes were connected at the inlet and outlet channels. Finally, the setup was completed by assembling the tubing and connected to a syringe pump AcTIVA A22 (ADOX, Ituzaingó, Argentina).

### 2.2. Computational Fluid Dynamics Characterization 

COMSOL Multiphysics version 5.2a software was used to predict fluid dynamic profiles in the microfluidic device design. An inlet flow rate of 600 µL/min was evaluated as the experimental relevant condition. The computational calculation was based on the Navier–Stokes equations, and due to the very low Reynolds number in microfluidic devices, the inertial component was neglected, allowing a higher mesh density for computation. The 3D model boundary conditions included the water properties for the flowable materials, progressive laminar flow, zero pressure at the outlet, and no-slip. In addition, >900,000 freedom elements (average element quality of >0.6) were included for the construction of the mesh density. The methodology was modified from [25]. 

### 2.3. Cell Culture in the LM Bioreactor

The Chinese hamster ovary CHO-ahIFN-α2b adherent cell line was used [25] to evaluate the production of recombinant antibodies in the MM bioreactor. This cell line expresses the anti-hIFN-α2b recombinant scFv-Fc and was obtained from Attallah et al., at Centro Biotecnológico del Litoral (UNL/CONICET) as previously described [34]. The CHO-ahIFN-α2b adherent cells were cultured in T-flasks with DMEM/F-12 growth media (Gibco, Waltham, MA, USA) supplemented with 5% (*v/v*) heat-inactivated fetal bovine serum (FBS) (Agropharma, Moreno, Argentina), 50 mg/mL gentamicin (Invitrogen, New York, NY, USA) and 2.44 g/L NaHCO_3_ at 37 °C in 5% CO_2_. The medium for antibody production was the same, but supplemented with 0.5% (*v/v*) FBS.

Before seeding the cells in the LM bioreactor, the surface of the microchannels was exposed to oxygen plasma for 3 min to increase PDMS surface hydrophilicity. Then, 70% (*v/v*) ethanol was injected into the microchannels inlet, and the excess volume was removed at the outlet with a syringe. After that, the LM bioreactor was disinfected using 0.5 M NaOH for 30 min and then rinsed with sterile water under sterile conditions. Before cell seeding, the chip was incubated with 3 mL of a sterile solution with 0.1 mg/mL poly-D-lysine hydrobromide (Sigma, Saint Louis, MO, USA) for 30 min at 37 °C to enhance cell adhesion [28]. 

To seed the CHO-ahIFN-α2b cells into the LM bioreactor, they were resuspended from the T-flask with 0.5 mg/mL trypsin and 0.2 mg/mL EDTA-Na_4_ (Gibco, New York, NY, USA), and incubated at 37 °C for 3 min. Trypsin was then inactivated with 10% (*v/v*) FBS, the cells were washed with phosphate buffer solution (PBS: 2.96 mM NaH_2_PO_4_, 1.06 mM KH_2_PO_4_, 155 mM NaCl, pH 7.4) and centrifuged at 1000× *g* rpm for 5 min. The cells were harvested and resuspended in fresh media, and cell number and viability were quantified using a Neubauer chamber and trypan blue exclusion (Invitrogen, Grand Island, NY, USA). Then, the CHO-ahIFN-α2b cells in the suspension were injected with a syringe and a needle into the inlet of the LM bioreactor for seeding at a concentration of 10^6^ cells per mL. The microfluidic device was incubated at 37 °C in a humidified incubator with 5% CO_2_ fed with growth culture media for 7 days until cell adhesion and confluence were achieved on the floor of the chip. After that, the cells in the device were fed every one or two days with the production culture medium for 11 days, and the culture medium was removed and stored at −20 °C for glucose, lactate, and antibody analysis. 

The adhesion of the CHO-ahIFN-α2b cells to the substrate and their growth in the microfluidic device was followed by standard imaging using bright-field microscopy. The microchannels were monitored using an inverted Olympus microscope CKX41 (Olympus, Tokyo, Japan). Bright-field images were acquired using an Olympus objective PlanC N 10X/0.25 with an Olympus QColor 5 camera and processed using QCapture Pro 6.0 software. Three micrographs per microchannel at a 10× magnification was acquired every day during the experiment to monitor their viability.

### 2.4. Quantification of Glucose and Lactate in the Culture Medium 

The concentration of glucose in the culture medium was quantified during the 18 days of the experiment in the LM bioreactor and the T-flask using the Accu-Chek Performa meter (Roche, Germany). A sample of at least 0.6 μL of culture media was placed on the Accu-Chek Performa test strip to make the measurements. As a baseline control, the concentration of glucose in the fresh growth culture medium and production medium was measured. 

In addition, the concentration of lactate in the culture media was quantified with a Lactate kit (Wiener Lab, Rosario, Argentina) following the manufacturer’s instructions. In this method, the lactate in the samples is oxidized by the specific enzyme lactate oxidase. The hydrogen peroxide formed in this reaction is then used by peroxidase to generate a chromogen. The color intensity of the complex formed is directly proportional to the concentration of L-lactate in the sample and was determined by measuring the absorbance at 545 nm in a Multiskan MCC/340 multi-well plate reader (Labsystems, Helsinki, Finland). The protocol for the samples from the different culture conditions was performed in a 96-well plate. A Calibrator A-plus standard was processed in the same way as the samples to calculate the concentration factor. 

### 2.5. ELISA Quantification of Anti-hIFN-α2b 

To quantify the concentration of anti-hIFN-α2b produced under the different culture conditions, we performed indirect ELISA by measuring absorbance at 492 nm in 96-well plates (Greiner Bio-One, Kremsmünster, Austria) in a multi-well plate reader (Labsystems Multiskan MCC/340, Finland) as previously described in Bourguignon et al., [25]. Between each step, plates were washed 6 times with 200 µL/well PBS containing 0.05% (*v/v*) Tween 20 (PBS-T). Samples and antibody dilutions were prepared in PBS-T with 0.1% (*w/v*) BSA. A 1:2 serial dilution curve from 1000 ng/mL to 7.8 ng/mL of protein A affinity purified mAb was used as a standard. The assay was reproduced in triplicate. A competitive ELISA described by Friguet et al. [32] and modified by the bivalent effect of the antibodies [35] was used to determine the affinity constants (K_A_) of the complexes produced between rhIFN-α2b and mAbs [36].

### 2.6. Inhibition of the Antiproliferative Activity of rhIFN-α2b by Anti-hIFN-α2b

The antiproliferative activity of rhIFN-α2b was determined by its ability to inhibit Daudi cell growth [25,37,38]. In contrast, the neutralizing effect of the mAb was evaluated as the ability to allow cell growth. The methodology was previously described in [25]. Cell proliferation was determined using CellTiter 96^TM^ AQueous, a non-radioactive cell proliferation assay (Promega, Madison, WI, USA). The microplate reader was used to determine absorbance values at 492 nm and dose-response curves were plotted against mAb concentrations to determine the IC_50_. The IC_50_ is defined as the mAb concentration required to inhibit 50% of the maximal antiproliferative activity induced by rhIFN-α2b. The assay was reproduced in triplicate. The concentration of mAbs in the supernatants was determined by a specific indirect ELISA. For that, a 1:2 serial dilution curve from 2500 ng/mL to 19.5 ng/mL of protein A-affinity purified anti-rhIFN-α2b was used as a standard.

### 2.7. Determination of Affinity Constants of Anti-hIFN-α2b

For the competition, different concentrations of soluble rhIFN-α2b were preincubated overnight at room temperature with a constant amount of mAb. Then, an aliquot of the mixture was incubated for 2 h at 37 °C in 96-well plates coated with 50 ng per well of the cytokine diluted in bicarbonate buffer (50 mM, pH 9.6) followed by PBS-BSA blocking solution. After washing 6 times with PBS-T, bound mAbs were detected using suitably diluted polyclonal rabbit anti-human immunoglobulins (DAKO, Glostrup, Denmark) for 1 h at 37 °C, followed by an appropriate dilution of peroxidase-labeled, goat, anti-rabbit immunoglobulin (DAKO, Glostrup, Denmark). Enzyme conjugates were diluted appropriately with 0.1% (*w/v*) PBS-T with BSA (PBS-T-BSA) and incubated for 1 h at 37 °C. Plates were washed 6 times with PBS-T and incubated in the dark with 50 mM citric acid phosphate buffer, pH 5.3, containing 3 mg/mL o-phenylenediamine and 0.12% (*v/v*) H_2_O_2_ (substrate solution), and the reaction was stopped with 2N H_2_SO_4_. Absorbance was measured at 492 nm with the plate reader. The assay was reproduced in triplicate. The affinity constant was determined by Scatchard plot analysis.

### 2.8. Inhibition of rhIFN-α2b Cell Signaling by Anti-hIFN-α2b 

To assess the inhibition of cell signaling activated by rhIFN-α2b, HeLa Mx2/eGFP reporter cells were seeded in 96-well plates (10^4^ cells/well) and incubated for 24 h at 37 °C with 5% CO_2_. This cell line was developed at the Centro Biotecnológico del Litoral, Facultad de Bioquímica y Ciencias Biológicas (UNL) by Bürgi et al., as previously reported [39], introducing the reporter gene eGFP (enhanced green fluorescence protein) under the control of the Mx2 promoter, considering that Mx genes are rapidly and specifically induced by type I IFNs (IFN-I). HeLa cell culture supernatants were discarded and 1:2 serial dilutions of supernatants from microfluidic devices or T-flask containing anti-rhIFN-α2b were added and preincubated for 2 h at 37 °C with a volume equal to 50 U/mL of rhIFN-α2b. Cells were then incubated for 24 h at 37 °C with 5% CO_2_. Cells were trypsinized, carefully suspended in 0.2 mL PBS, and then eGFP expression was measured by flow cytometry. The concentration of mAbs in the supernatants was determined by a specific indirect ELISA as mentioned before. Flow cytometry was performed in a Guava^®^ Easy Cyte^TM^ cytometer (Millipore Sigma, Hayward, CA, USA). This cytometer has a 488 nm blue laser to access commonly used fluorescent dyes and detectors to measure five different parameters: 3 fluorescent channels and the 2 light scatters, lateral (SSC), and direct (FSC). This kit can also measure samples from 96-well plates. Data acquisition and analysis were performed with Guava CytoSoft^TM^ 3.6.1 software. For each sample, 5000 events were collected in the FSC vs. SSC dot plot gating. Flow calibration and optical alignment were performed with the help of flow control Fluorospheres (Guava^®^ Check Millipore Sigma kit, Hayward, CA, USA) before each determination. Cells were tested for eGFP signal (meaning fluorescence intensity times the percentage of eGFP positive cells), which is highly proportional to mRNA levels [40]. The ratios between the mean absorbance of wells incubated in the presence of each preincubated mAb-cytokine and the mean absorbance of wells incubated only in the presence of the cytokine were determined. These ratios were plotted on dose-response curves against mAb concentrations. The potency of anti-rhIFN-α2b was determined as the concentration of mAb required, that produced 50% inhibition of maximal fluorescence using HeLa Mx2/eGFP (FRC_50_) cells induced by rhIFN-α2b. Assays were performed in triplicate and repeated twice.

### 2.9. Inhibition of the Antiviral Activity of rhIFN by Anti-hIFN-α2b 

To evaluate the antiviral activity of rhIFN-α2b, an assay based on the MDBK cell line obtained from ATCC (ATCC CCL-22) challenged with the vesicular stomatitis virus (VSV) Indiana strain obtained from ATCC (ATCC VR-158) was used. Briefly, MDBK cells were grown overnight in 96-well plates (2.5 × 10^4^ cells/well). The next day, serial 1:2 mAb dilutions from the microfluidic devices or T-bottles were preincubated for 2 h at 37 °C with an equal volume of 4 U/mL rhIFN-α2b and added to each well containing the cells. After an incubation period of 6 h, the supernatant was removed and an appropriate VSV suspension (generating a 100% cytopathic effect after 24 h) was added to each well. After overnight incubation, viable cells were measured by the crystal violet staining method. The mean absorbance of the ratio (antibody-preincubated cytokine/free cytokine) versus mAb concentration was plotted on a dose-response curve, and the potency of each sample was calculated. Potency was determined as the concentration of the mAb that inhibits 40% of the antiviral activity (IC_40_) induced by rhIFN-α2b. Assays were replicated thrice (intra-assay variation) and repeated twice (inter-assay variation).

### 2.10. Statistical Analysis

The experimental data were stated as the mean standard deviation (SD) from at least three experiments. One-way analysis of variance (ANOVA) was used for statistical analysis, *p*-values of less than 0.05 were considered statistically significant (*p* < 0.05).

## 3. Results and Discussion

### 3.1. LM Bioreactor Manufacturing

**Figure 2:**
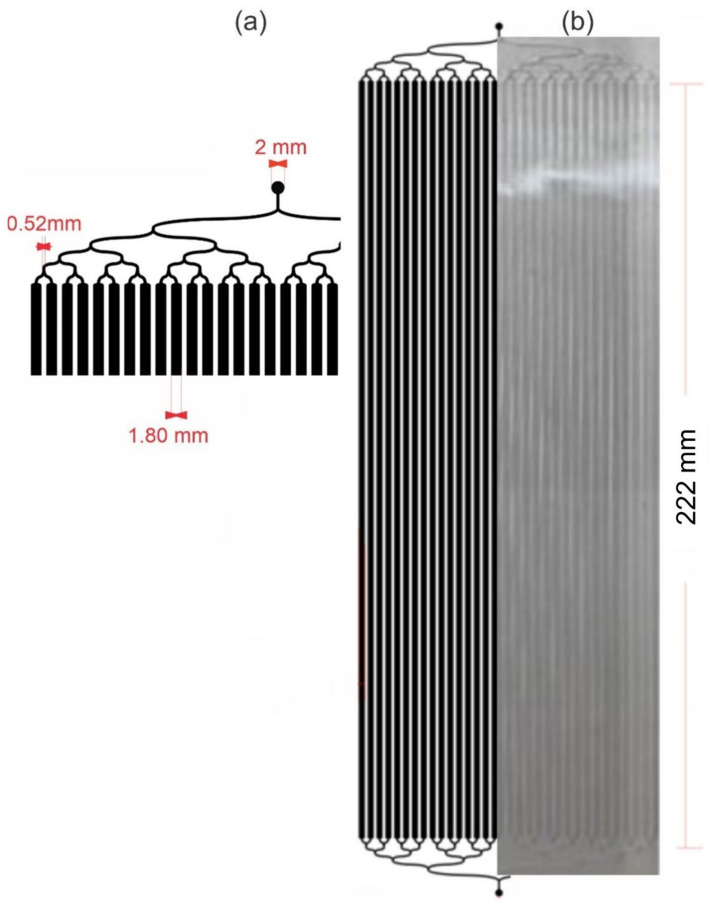
LM bioreactor used in this study. (**a**) Design of microchannels with one inlet, one outlet, and 32 cell culture cisterns. (**b**) Polydimethylsiloxane/glass LM bioreactor with a 182 cm^2^ surface area, height of 200 µm, and a total internal volume of 2.8 mL.

In previous studies, we demonstrated the good performance of microfluidic devices to produce mAbs [24], therefore in this work we scaled up the microfluidic device, increasing the number of channels and length, and in this way raised the total volume of the device, maintaining the dimension of the microchannels (Figure 2). This PDMS-Glass LM bioreactor with 32 cisterns was designed with a 184 cm^2^ surface area and a volume of 2.8 mL. The device consists of an inlet channel that branches off five times, connecting in parallel to 32 cisterns 22.20 mm long through which the culture medium flows, that converge symmetrically in pairs into a single outlet channel (Figure 2). 

The inlet and outlet flow channels were designed symmetrically with curved edges and no 90° angles to simplify the loading of the cells and the flow of the culture medium. The main body of the device with long, gently curved cisterns without inter-cistern connectors was designed to favor cell distribution in the microfluidic device with more homogeneous flows within the channels. 

The washing, culture medium exchange, and cell seeding were performed automatically with a syringe pump, a method that is frequently used in microfluidic devices [41,42,43,44,45]. In this way, the microfluidic cell culture systems are constantly perfused with the cell culture medium. The constant perfusion of microfluidic culture devices ensures that nutrients are continuously supplied, and waste products are removed. A flow rate of 300 μL/min for 10 min was used, and the LM bioreactor volume was renewed every 24 h in a sterile closed circuit. 

These operations of addition and withdrawal of media, are performed carefully, handling equivalent amounts of media at the inlet and outlet to minimize convective flow effects. The size of the systems allows for lower consumption of reagents than conventional devices, and this property makes them ideal candidates when the cost of reagents is a limiting factor. With a single surface of 182 cm^2^, this device is the first of this size to be made with the F-Mold technology, showing the feasibility of producing large-surface microfluidic devices with this innovative methodology.

### 3.2. Computational Modeling of the Flow in LM Bioreactor

**Figure 3:**
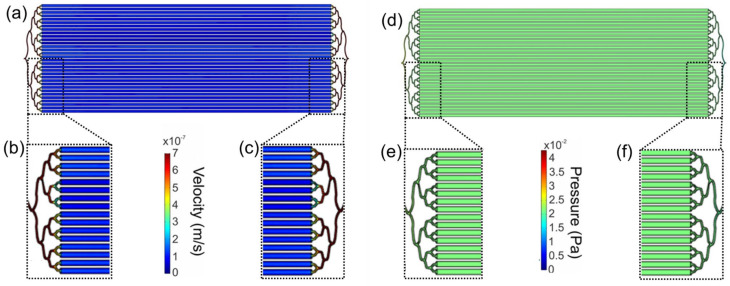
Computational modeling of the flow in the large-area microfluidic bioreactor. Variable velocity (m/sec) in (**a**) the whole design (**b**) inlet and (**c**) outlet of the chip. Pressure (Pa) in (**d**) the whole design, (**e**) inlet and (**f**) outlet of the chip. The simulations were created in the microchannels at a height of 75 μm. Figures generated from an arbitrary input stream 1 × 10^−8^ m^3^/s (600 µL/min).

A discontinuous pumping method with the flow every 24 h in the closed microfluidic system using a pump pressure syringe ADOX-AcTIVA A22 for the bioprocess in the LM bioreactor. A computational simulation of the flow inside the LM bioreactor was performed to predict whether the medium will flow homogeneously throughout the design and to know if the numerical conditions will affect cell growth. The flow was computationally modeled within the LM bioreactor from an arbitrary input stream of 600 µL/min. The calculated flow velocities within the LM bioreactor show areas of reduced flow of 10^7^ m/s (blue areas) in the cisterns (Figure 3a) and higher velocity values in the inlet (Figure 3b.) and the outlet (Figure 3c) of the design. The laminar flow in the LM bioreactor ensures diffusive mass transport that allows the generation of a continuous, homogeneous cell monolayer [46].

The pressure value calculated for the cisterns was Pa ~0.02 (Figure 3d). The cisterns of the device showed areas of reduced pressure (green areas). Higher pressure areas (yellow to red, Figure 3e) are in the inlet/entrance of the microchannels, and lower pressure (light blue to blue, Figure 3f.) was identified in the outlet/exit of the channels. The values calculated are related to those reported in the physiological environment of the human ovarian epithelium [47].

These results suggest that the configuration of the microchannels in the LM bioreactor provides a laminar flow velocity profile, and it is reduced in the cisterns. The flow rate in the cisterns 14 to 16 at the center of the device is reduced as compared with others; this may be due to noise in the model. It results in a suitable microenvironment for the growth and proliferation of adherent cells in long-term discontinuous pumping cycles of 24 h. Computer modeling of the flow within the LM bioreactor would provide evidence that this would be a suitable method for growing adherent cells. Also, the CHO cells seeded and cultured in the LM should be able to tolerate the pressure exerted inside the device [48], and it would not be expected to significantly affect the production of recombinant proteins in it.

### 3.3. Cell Growth in the LM Bioreactor and Glucose/Lactate Quantification

**Figure 4:**
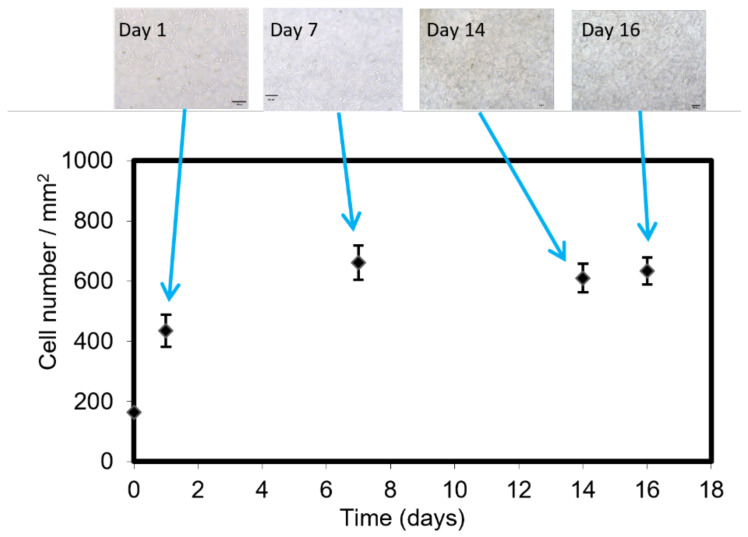
Cell density in large-area microfluidic bioreactor. On day 7, the growth culture medium was changed to the production medium. Image magnification: 20×, Bar: 100 μm. Error bars indicate standard error of the mean determinations.

The adherent CHO-ahIFNα2b cell line expressing anti-hIFN-α2b was selected to cultivate in the LM bioreactor and study its proliferation in long-term culture by analyzing the images obtained by bright-field microscopy. The poly-D-lysine coated LM bioreactor was seeded with the cells at an initial concentration of 10^6^ cells/mL and cell adhesion was observed on the floor of the LM bioreactor. The images obtained from day 1 of culture indicate that the morphology of most of the cells was fusiform with the appearance of borders that suggest a strong adhesion to the LM bioreactor floor, reaching a monolayer on day 7 of culture, and remaining constant for the following 11 days. The total incubation time was 18 days (Figure 4). The maximum cell number in the LM bioreactor was 670 cells/mm^2^ and remained constant over time. On day 7, the growth culture medium with 5% (*v/v*) FBS was changed to the production medium with 0.5% (*v/v*) FBS, and samples were collected from the output of the device to determine the concentration of functional antibodies by specific indirect ELISA.

The morphology of most cells was fusiform, with the appearance of borders that suggest a strong adhesion to the floor of the LM bioreactor [49], reaching 18 days of culture, a longer culture time than has been previously reported in the literature for microfluidic devices [28,45,50,51,52,53].

**Figure 5:**
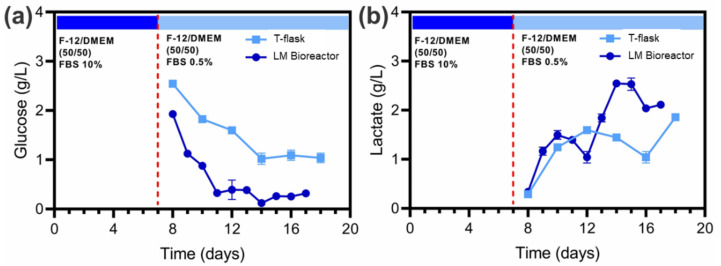
The concentration of (**a**) glucose and (**b**) lactate in T-flasks and large-area microfluidic bioreactor during the production phase from day 8 to 17. Error bars indicate standard error of the mean determinations.

Glucose and lactate levels of the culture medium in traditional adherent cell cultures (T-flasks) and the LM bioreactor were quantified (Figure 5). In the T-flasks on day 8, the glucose concentration was 2.55 g/L and decreased until reaching 1.04 g/L on day 18 (Figure 5a), while the lactate concentration was less than 0.3 g/l on day 8 until reaching 1.86 g/L on day 18 (Figure 5b). On the other hand, the glucose concentration in the LM bioreactor was 1.93 g/L on day 8 and decreased to 0.32 g/L on day 17 (Figure 5a), while the lactate concentration was 0.34 g/L on day 1, and it increased to a maximum of 2.55 g/L on day 15 (Figure 5b).

Mammalian cells are the preferred expression system for therapeutic antibodies because the cellular machinery for glycosylation is similar to that of human cells [54]. On the other hand, previous works reported that the availability of glucose in the culture medium can affect the glycosylation of an antibody secreted by CHO cells [55,56]. In addition, periodic intervals of glucose depletion of the culture medium during fed-batch cultures lead to the production of antibodies with low levels of glycosylation or directly non-glycosylation [55]. Liu et al. [55] also observed a strong positive correlation between the length of time cells were exposed to glucose depletion and the proportion of non-glycosylated antibodies. Moreover, there are clear relationships between the structure and function of antibody glycans [57], so it is important to control the factors that are critical for antibody glycosylation in a bioprocess to ensure the desired function. In particular, it has recently been shown that differences in the glycosylation of the Fc region of anti-hIFN-α2b tend to modify the in vitro neutralization capacity of this particular molecule [58]. Therefore, the glucose and lactate levels of the CHO-ahIFNα2b culture media over time were quantified in the LM bioreactor and T-flask in parallel. We observed that the glucose concentration in LM bioreactor culture media remains low relative to the T-flasks cultures (Figure 5). In contrast, the lactate concentration was higher after 14 days of culture time in the LM bioreactor. This could be expected because during cell growth, higher consumption of glucose, and production of lactate over time in the LM bioreactor was observed to have a medium volume of 2.8 mL, which is much less than the final volume of 10 mL in the T-75 flask. In turn, the surface for cell growth in the LM bioreactor is 184 cm^2^ (higher area/volume ratio), compared to the 75 cm^2^ of the T-75 flask. The relatively low levels of glucose could explain a differential metabolic stress in the cells that could affect the glycosylation of the antibody and its functional activity.

### 3.4. mAb Production in the LM Bioreactor

**Figure 6:**
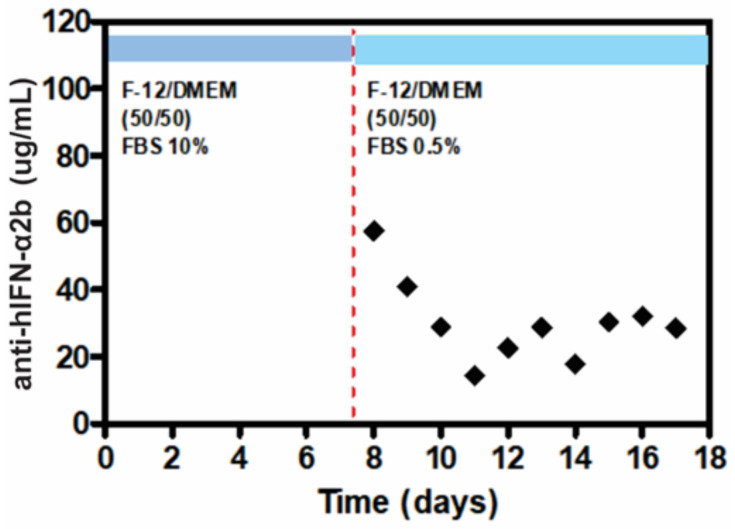
Concentration of anti-hIFN-α2b monoclonal antibody in supernatants of large-area microfluidic bioreactor culture from day 8 to day 18 of production is shown in black diamonds.

The production of anti-hIFN-α2b secreted by CHO-ahIFNα2b cells every 24 h for 18 days was evaluated (Figure 6). Initially, the concentration of anti-hIFN-α2b secreted by the cells was approximately 57 µg/mL, then mAb production decreased until day 12, when the cell line reached a plateau of anti-hIFN-α2b expression showing 32 µg/mL until the end of the culture on day 18. During the production phase, stressed cell signals were observed in the morphology of the cells, which was expected due to the reduced concentration of FBS in the production culture medium. This can affect cell growth and proliferation, redirecting cell metabolism primarily to antibody production. 

The affinity constant (K_A_) of anti-hIFN-α2b produced in the LM bioreactor was compared to that of the mAb produced in T-flask to analyze whether the culture conditions could affect the ability of the mAbs to bind to the cytokine. Surprisingly, in the LM bioreactor it was possible to produce antibodies that have a higher affinity constant compared to those produced in T-flasks. The anti-hIFN-α2b produced in the LM bioreactor presented K_A_ values ~2 times higher than the mAb produced in T-flasks (*p* <0.05, Table 1). The mAbs exhibited a significantly higher binding capacity when they and the cytokines were in the liquid phase under experimental conditions. One possible explanation for what is observed here is that epitope binding of the mAb may be affected by antibody glycosylation, which, in turn, may vary by glucose availability [58].

### 3.5. Characterization of the Inhibition of Antiproliferative Activity, Inhibition of Cell Signaling, and Inhibition of the Antiviral Activity of rhIFN-α2b by Antibodies Produced in LM Bioreactor and T-Flasks

**Figure 7:**
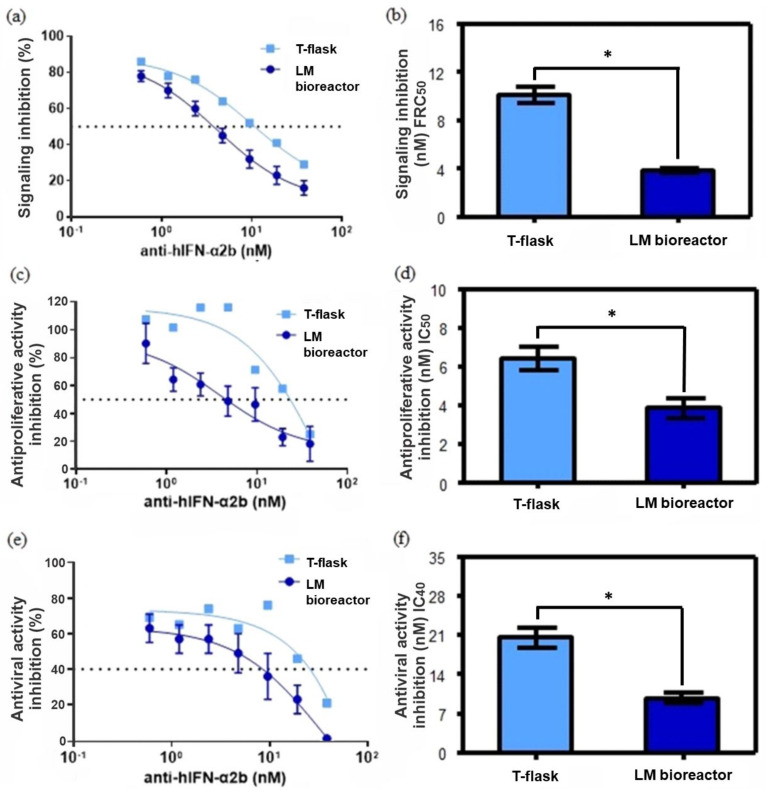
Evaluation of the neutralizing capacity of the biological activity of the anti-hIFN-α2b produced in LM bioreactor and T-flask. (**a**) Dose-response curve of the rhIFN-α2b signaling inhibition with increasing concentrations of the mAb produced in the LM bioreactor and T-flask. (**b**) Inhibition was measured as the concentration of mAb necessary to reduce 50% of fluorescence (FRC_50_) using HelaMx2/eGFP cells. (**c**) Dose-response curve of the rhIFN-α2b antiproliferative activity inhibition with increasing concentrations of the mAb produced in LM bioreactor and T-flask. (**d**) Inhibition was measured as the concentration of mAb necessary to reduce 50% of the maximum cytokine potency (IC_50_) using Daudi cells. € Dose-response curve of the antiviral activity inhibition of rhIFN-α2b with increasing concentrations of the mAb produced in the LM bioreactor and T-flask. (**f**) The rhIFN-α2b antiviral activity inhibition was measured as the concentration of mAb necessary to reduce 40% of the maximum cytokine potency (IC_40_). The error bar represents the standard error of the mean. The presence of asterisks (*) denotes significant differences determined by Tukey’s test *p* < 0.05, than those calculated for the mAb produced in the T-flask, determined by ANOVA followed by Tukey’s test.

Since production in the LM bioreactor significantly enhances anti-hIFN-α2b binding capacity in terms of higher K_A_, other mAb quality attributes tested were related to its biological activity. In particular, the ability to inhibit the antiproliferative activity of rhIFN-α2b, rhIFN-α2b signaling, and the ability to inhibit the biological antiviral activity of rhIFN-α2b by the action of the mAb produced in LM bioreactor by CHO-ahIFNα2b cells were evaluated and compared to those produced, by the same cell line, grown and adhered to the bottom of the T-flask (Figure 7). The ability of the mAb to inhibit rhIFN-α2b signaling was determined by fluorescence analysis using indicator cells whose eGFP expression is mediated by the rhIFN-α2b-inducible Mx promoter. Figure 7a shows the signaling inhibition dose-response curves corresponding to the concentration of the mAb needed to reduce 50% of the fluorescence (FRC_50_) measured by flow cytometry. Antibodies produced by the cell line using T-flask culture exhibited significantly higher FRC_50_ than LM bioreactor (Figure 7d, *p* < 0.05; Tukey’s test), this means that the rhIFN-α2b neutralizing capacity of the mAbs produced in the LM bioreactor was greater than that of the mAbs produced in T-flask.

Also, the concentrations of mAbs necessary to inhibit 50% of the antiproliferative activity of the cytokine (IC_50_) were determined (Figure 7c,d). As in the previous experiment, the IC_50_ for the mAb from the LM bioreactor was significantly lower (*p* < 0.05). 

Finally, the ability of anti-hIFN-α2b to inhibit the antiviral biological activity of rhIFN-α2b was also investigated. The antiviral activity of rhIFN-α2b was significantly less affected (*p* < 0.05) by the T-flask-produced mAbs than those produced by the LM bioreactor (Figure 7e,f), supporting the idea of a strong correlation between the mAb affinity and the neutralizing action.

Taken together, these results show that the LM bioreactor carrying out production, significantly improves the biological functions of the anti-hIFN-α2b antibody in terms of its cytokine binding and neutralization capacity, compared to those produced by the traditional adherent cell culture method. These results should be viewed with caution, as the neutralizing activity depends on the affinity of the mAb for an epitope that belongs to the cytokine area that binds to the receptor. Furthermore, for antibodies that recognize a common epitope, it is assumed that their increasing affinity is related to their increasing neutralization. In this case, the anti-hIFN-α2b with the highest affinity for rhIFN-α2b showed superior neutralizing activity.

The same molecules produced by the same mammalian cells in different culture methods demonstrated different capacities to neutralize rhIFN-a2b. Neutralizing activity depends on both the affinity of the antibody and the ability of the antibody to block a cytokine-receptor-binding area. Previously, it has been reported that structural differences in the domains of the heavy chain constant regions of antibodies are responsible for differences in affinity constants and, consequently, for variations in antigen binding [59,60]. The mechanism of this effect could involve the transformation of the binding site structure into a kinetically more competent mAbs with higher binding affinities [61]. Considering that anti-hIFN-α2b has N-glycosylation sites in its Fc region, glycosylation can be affected by the culture conditions in terms of glucose availability and this, in turn, can affect mAb binding as was demonstrated by Attallah et al., 2020 [58]. Future studies focused on the characterization of the anti-hIFN-α2b glycosylation are planned. 

## 4. Conclusions

In this article, we show the development of a large-area microfluidic bioreactor that allows the culture of mammalian cells to scale up the production of mAbs compared to earlier microfluidic devices [25]. The device design with 32 cisterns ensures a homogeneous environment regarding flow velocity and pressure, as predicted from the CFD simulations. The fabrication process used here is low-cost and allows the obtaining of large-size microfluidic devices. The LM bioreactor provided highly controlled cell culture conditions in a confined microenvironment with reduced levels of glucose, which may improve the quality of mAbs produced, with potential applications for the production of recombinant proteins for drug screening or early stages of drug discovery.

The anti-hIFN-α2b produced by the CHO-ahIFN-α2b cell line in the LM bioreactor presented K_A_ values 2 times higher than the anti-hIFN-α2b produced by the same cell line in the T-flask. Moreover, the biological function of cytokine neutralizing capacity in terms of antiproliferative activity, signaling, and antiviral activity by the anti-hIFN-α2b produced in the LM bioreactor were significantly higher compared to the mAb produced with the standard T-flask adherent cell culture method. The results obtained here validate the functionality of the novel technology proposed and confirm that the LM bioreactor could be a valuable alternative tool to develop new bioprocess strategies to produce high-quality biologics. Additionally, the microsystem we developed allows the detailed evaluation of multiple factors influencing cell culture (biochemical factors, flow rate, and inoculum concentration), suggesting that it might be useful in novel biotherapeutics discovery and process development. 

To the best of our knowledge, this is the largest area PDMS microfluidic device ever made for mammalian cell culture. We show that it is possible to culture mammalian cell lines in these devices and that they can be used to produce recombinant proteins with potential for therapeutic use. Moreover, this is the first time that the recombinant monoclonal antibody produced in a microfluidic device exhibits better functional activity than the antibody produced under standard culture conditions. In this way, we demonstrate that the platform developed can illuminate new strategies of culture process development to better produce biotherapeutics.

## Figures and Tables

**Table 1:** Anti-hlFN-α2b affinity constant (K_a_) evaluation in T-flask and LM Bioreactor.

Culture condition	K_a_(10^9^M^−1^)
T-flask	1.8 ± 0.1 *
LM Bioreactor	3.6 ± 0.3 *
Ratio LM Bioreactor/T-flask = 2	
* Significant differences (*p* < 0.05) determined by Student’s *t*-test	

## Data Availability

Not applicable.
